# Towards the rate limit of heterologous biotechnological reactions in recombinant cyanobacteria

**DOI:** 10.1186/s13068-022-02237-4

**Published:** 2023-01-06

**Authors:** Giovanni Davide Barone, Michal Hubáček, Lenny Malihan-Yap, Hanna C. Grimm, Lauri Nikkanen, Catarina C. Pacheco, Paula Tamagnini, Yagut Allahverdiyeva, Robert Kourist

**Affiliations:** 1 Biocatalysis and Protein Engineering, Institute of Molecular Biotechnology, Graz University of Technology, 8010 Graz, Austria; 2 i3S - Instituto de Investigação e Inovação em Saúde, IBMC - Instituto de Biologia Molecular e Celular, Universidade do Porto, 4200-135 Porto, Portugal; 3 Departamento de Biologia, Faculdade de Ciências, Universidade do Porto, 4169-007 Porto, Portugal; 4 Laboratory of Molecular Plant Biology, Department of Life Technologies, University of Turku, 20014 Turku, Finland

**Keywords:** Biotransformations, Cyanobacteria, d-Glucose, *Synechocystis* sp. PCC 6803, Ene-reduction

## Abstract

**Background:**

Cyanobacteria have emerged as highly efficient organisms for the production of chemicals and biofuels. Yet, the productivity of the cell has been low for commercial application. Cyanobacterial photobiotransformations utilize photosynthetic electrons to form reducing equivalents, such as NADPH-to-fuel biocatalytic reactions. These photobiotransformations are a measure to which extent photosynthetic electrons can be deviated toward heterologous biotechnological processes, such as the production of biofuels. By expressing oxidoreductases, such as YqjM from *Bacillus subtilis* in *Synechocystis* sp. PCC 6803, a high specific activity was obtained in the reduction of maleimides. Here, we investigated the possibility to accelerate the NAD(P)H-consuming redox reactions by addition of carbohydrates as exogenous carbon sources such as D-Glucose under light and darkness.

**Results:**

A 1.7-fold increase of activity (150 µmol min^−1^ g_DCW_^−1^) was observed upon addition of D-Glucose at an OD_750_ = 2.5 (DCW = 0.6 g L^−1^) in the biotransformation of 2-methylmaleimide. The stimulating effect of D-Glucose was also observed at higher cell densities in light and dark conditions as well as in the reduction of other substrates. No increase in both effective photosynthetic yields of Photosystem II and Photosystem I was found upon D-Glucose addition. However, we observed higher NAD(P)H fluorescence when D-Glucose was supplemented, suggesting increased glycolytic activity. Moreover, the system was scaled-up (working volume of 200 mL) in an internally illuminated Bubble Column Reactor exhibiting a 2.4-fold increase of specific activity under light-limited conditions.

**Conclusions:**

Results show that under photoautotrophic conditions at a specific activity of 90 µmol min^−1^ g_DCW_^−1^, the ene-reductase YqjM in *Synechocystis* sp. PCC 6803 is not NAD(P)H saturated, which is an indicator that an increase of the rates of heterologous electron consuming processes for catalysis and biofuel production will require funnelling further reducing power from the photosynthetic chain toward heterologous processes.

**Supplementary Information:**

The online version contains supplementary material available at 10.1186/s13068-022-02237-4.

## Background

Cyanobacterial processes exploit photosynthetic water splitting and carbon dioxide fixation for the sustainable synthesis of chemicals and biofuels and could greatly alleviate land-use conflicts associated with the provision of the raw materials for a circular bioeconomy. Furthermore, cyanobacteria can be also applied for the catalytic upgrading and synthesis of high-value chemicals. Yet, biomass productivity and achievable space–time-yields have been too low for the successful production of biofuels and other bioproducts [1]. Current processes use approximately half of the fixed carbon for the production of organic molecules [2]. This shows to which extent the already existing production systems are pushing the photosynthetic metabolism to its limits. To assess the metabolic boundaries of an electron-consuming process in cyanobacteria, we investigated a nicotinamide adenine dinucleotide phosphate (NADPH)—or nicotinamide adenine dinucleotide (NADH)-dependent photobiocatalytic reaction in the cyanobacterium *Synechocystis* sp. PCC 6803 (hereafter *Synechocystis* or Syn). Photobiocatalysis has emerged as an interface between renewable energy and chemical synthesis. While light-driven enzymatic reactions [3, 4] and the coupling of photocatalysts with enzymes [5, 6] expand the catalytic scope of biocatalysis, the photobiocatalytic cofactor regeneration in cell-free [7] or cellular [8–10] systems has the potential to increase the efficiency of biocatalytic redox reactions. Current processes for the recycling of redox cofactors rely on auxiliary organic co-substrates, such as isopropanol or glucose. The oxidation of these co-substrates is highly problematic in terms of the atom economy of the process: one of the 12 principles of green chemistry [11]. The use of photosynthetic water-splitting as an electron source completely solves this problem and, additionally, avoids the formation of side-products such as acetone whose separation requires additional energy and cost. Whole cell photobiotransformations have been achieved by the expression of the genes of several oxidoreductases in cyanobacteria [12–15] with activities in the range of 26–60 µmol min^−1^ g_DCW_^−1^ (or U g_DCW_^−1^) [16, 17]. With up to 100–120 U g_DCW_^−1^_,_ the highest rates have been obtained so far in the stereoselective reduction of activated C=C double bonds by the ene-reductase YqjM from *B. subtilis* in *Synechocystis* [12]. The major limitation of photosynthetic production system is light penetration in dense cultures leading to the formation of photosynthetically inactive ‘dark zone’ and the loss of productivity. This is especially true in reactions utilizing high optical densities where the surface layers are already light-saturated while the inner layers are light-limited. Even though modern photobioreactors using internal illumination [18, 19] and other geometries with short light pathways [20] improve light supply and alleviate this to some extent, previous work has shown that whole cell cyanobacterial biotransformations cannot be performed with cell densities above a few grams per liter [21]. This makes it imperative to increase the enzyme’s specific activity in order to achieve competitive volumetric productivities and to identify potential bottlenecks for cell productivity.

Previously, we have shown that cyanobacterial photobiotransformation is limited by the supply of the reduced NADPH cofactor. Although the ene-reductase accepts NADH, it prefers NADPH as the main electron source. Stopped-flow kinetic measurements showed that in the stereoselective conversion of prochiral 2-methylmaleimide **1a** to *R*-2-methylsuccinimide **1b,** the reduction of the flavin mononucleotide (FMN) cofactor by the nicotinamide cofactor is the limiting step in the mechanism of YqjM [21]. The observed rates in darkness spurred our interest to which extent glycolytic pathways contribute to the observed specific activity and might increase reaction rates.

**Fig. 1:**
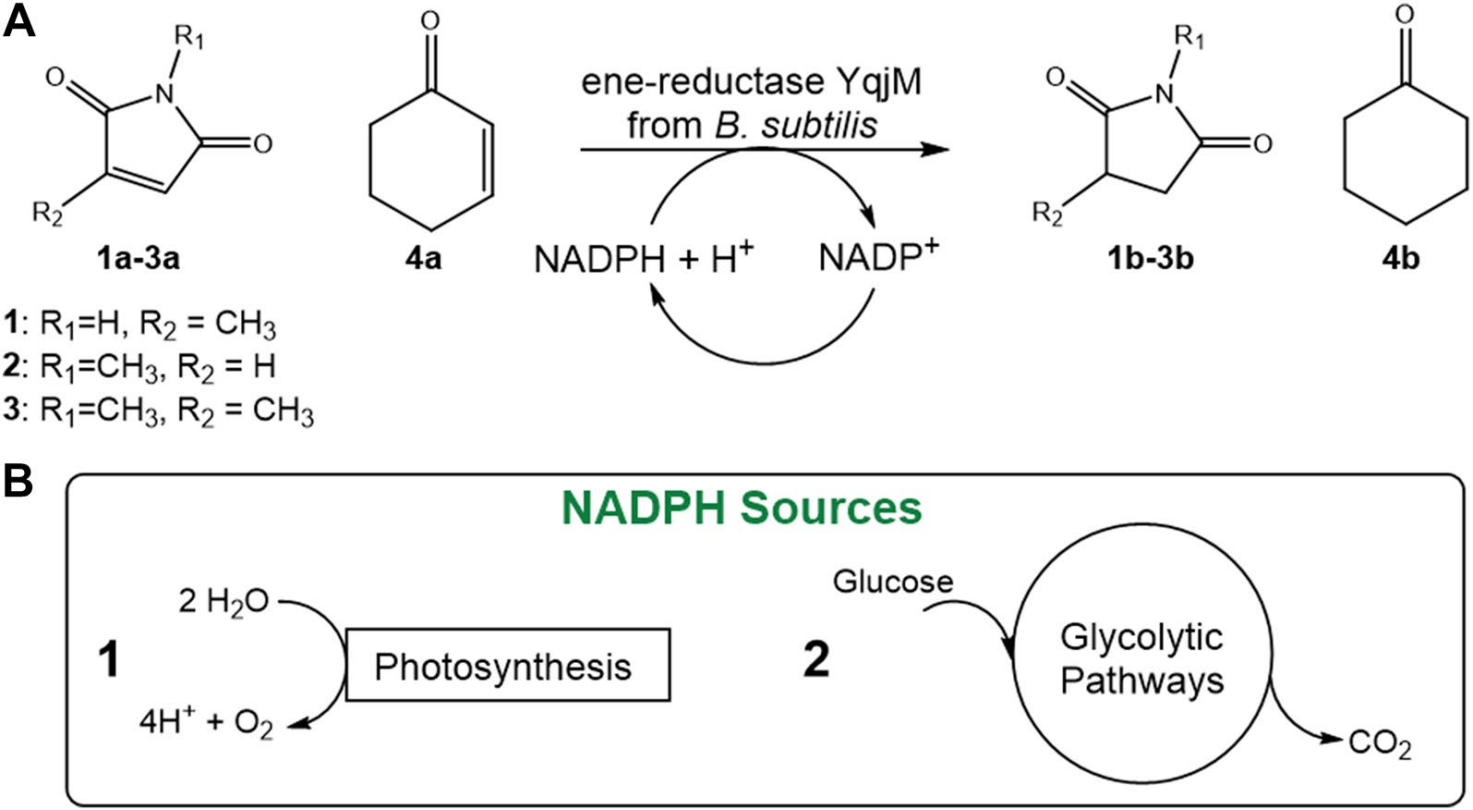
(**A**) C=C reduction catalysed by recombinant ene-reductase YqjM in *Synechocystis*; (**B**) Two possible sources of reductants in photosynthetic microorganisms and their use in intracellular cofactor recycling

Several photosynthetic organisms possess the ability to metabolise exogenous sources of organic carbon in dark (heterotrophy) or under light (mixotrophy). Mixotrophic culture under light and with a reduced carbon source in the medium leads to the co-action of light-dependent pathways and organic carbon metabolism and has been used to increase biomass accumulation and bioproduct yield [22]. The mixotrophic approach is usually justified when productivity and robustness of manufacturing should be significantly increased and when the approval of a pharmaceutical or nutraceutical product is at stake [23]. In this regard, *Synechocystis* is able to natively switch from photoautotrophy to mixotrophy [24], which significantly enhances its growth. Additionally, the cyanobacterial metabolism has a flexible response to additional electron demands [25]. Three pathways have been described to oxidize carbohydrates: (i) glycolysis, the Embden–Meyerhof–Parnas pathway (EMP); (ii) the oxidative pentose phosphate pathway (OPPp), which is highly active in cyanobacteria and whose oxidative part provides reduced NADPH; (iii) the Entner–Doudoroff pathway (ED), whose presence in cyanobacteria and plant plastids was only recently shown. The oxidation of carbohydrates is physiologically significant especially in day–night cycles, under mixotrophic and also under autotrophic conditions [26]. Under light, the OPPp generates two molecules of NADPH per molecule of glucose [26, 27]. The OPPp plays a dominant role in the catabolism of D-Glucose (D-Glu) and glycogen in mixotrophic and heterotrophic conditions and the increase of its intermediates at the steady state levels has been noted in mixotrophy [28]. Via the EMP, two ATP and two NADH molecules are formed per molecule of glucose. On the other hand, when D-Glu is metabolized via the ED, only one of each compounds (i.e. ATP, NADH and NADPH) are produced [26]. Moreover, the NADH generated in later catabolic steps (such as the oxidation of glyceraldehyde-3-phosphate and the reactions of the tricarboxylic acid cycle) can be used for NADP^+^-reduction via the pyridine nucleotide transhydrogenase complex PntAB [29]. The EMP seems to be beneficial in terms of energy, since it has a higher ATP yield than ED and OPPp [26]. Unlike the OPPp, the oxidative tricarboxylic acid (TCA) cycle has low efficiency in NADPH generation in *Synechocystis*. The OPPp dehydrogenase activity is repressed as the flow of the oxidative TCA cycle increases, avoiding NADPH overproduction [27]. While mixotrophic cultures are well-investigated, little is known about the influence of mixotrophic conditions on the reaction rate of heterologous redox reactions that represents an additional electron sink for the photosynthetic cell and are thus a representative for electron-consuming biosynthetic pathways. Figure 1 shows the C=C reduction catalysed by YqjM in *Synechocystis* as well as possible sources of reductants in photosynthetic microorganisms. To investigate a possible contribution of light-dependent and independent pathways on the photobiotransformation, we studied the effect of D-Glu addition on the reaction rate of the NAD(P)H-dependent, YqjM-catalyzed ene-reduction in the D-Glu-tolerant strain *Synechocystis* using various substrates. It should be noted that the enzymes of these glycolytic pathways are produced to some extent under photoautotrophic conditions, which made us confident that addition of D-Glu to a photobiotransformation should have an accelerating effect [30]. Moreover, we also investigated the applicability of D-Glu addition in a scalable reaction system utilizing a Bubble Column Reactor (BCR) internally illuminated by Wireless Light Emitters (WLE).

## Results

### Glucose supplementation enhances production rates of *Synechocystis*

**Fig. 2:**
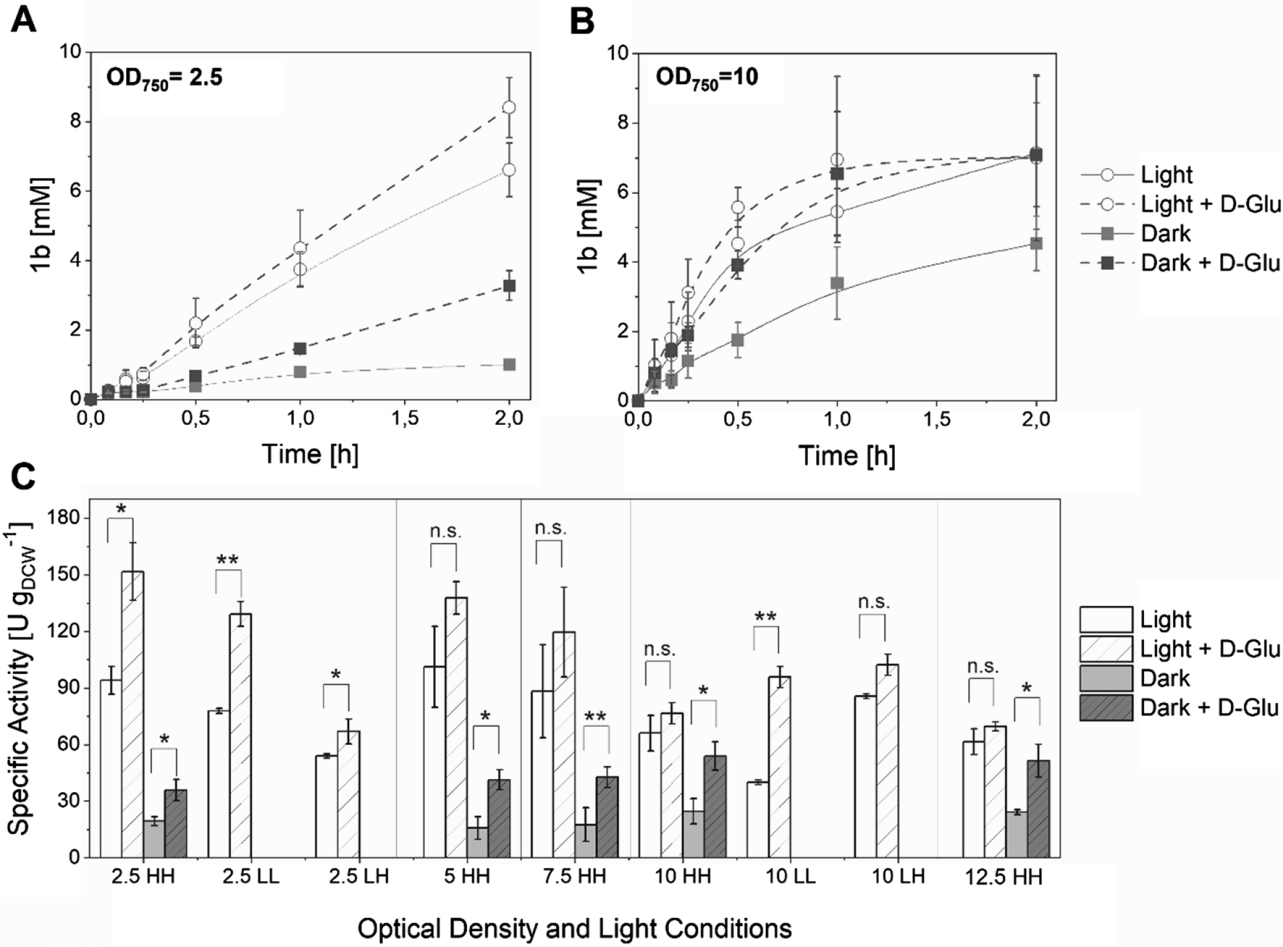
Whole-cell Biotransformation of **1a** mediated by Syn::P_*cpcB*_YqjM. Time course of product formation in BG-11 medium with and without addition of D-Glu (2.5 mM, added at the start of the reaction) at **(A)** OD_750_ = 2.5 corresponding to 0.6 g_DCW_ L^−1^ and at **(B)** OD_750_ = 10 corresponding to 2.4 g_DCW_ L^−1^. **C** Specific Activities from the reduction of **1a** at different optical densities and addition of D-Glu. HL = high light cultivation and low light reaction; LL = low light cultivation and reaction; HH = high light cultivation and reaction; LH = low light cultivation and high light reaction. *Reaction conditions:* V = 1.2 mL, T = 30 °C, 160 rpm, Initial concentration of **1a** = 10 mM, High light intensity = 200 µmol photons m^−2^ s^−1^, Low light intensity = 40–60 µmol photons m^−2^ s^−1^, *N* = 3. *P* values were calculated using Welch´s *t* test (*P ≤ 0.05; **P ≤ 0.005; *n.s*. = not significant) and correspond to the specific activity comparisons between reactions performed with and without D-Glu under light and darkness

To verify a possible contribution of glycolytic pathways to whole-cell biotransformations, we determined the rates of the same enzymatic ene-reduction with and without D-Glu under light and darkness. *Synechocystis* cells expressing the *yqjM* gene under the control of the strong promoter, P_*cpcB*_ (Syn::P_*cpcB*_YqjM), were cultivated under photoautotrophic conditions (that is, in the absence of D-Glu), harvested in the exponential phase (OD_750_ = 1–2) and then applied for whole-cell photobiotransformations. In previous works, a relatively high light intensity of approximately 150–200 µmol photons m^−2^ s^−1^ [12, 21] was identified to yield the highest specific activities in whole-cell biotransformations. Therefore, this light intensity was selected for the biotransformations. Moreover, the growth light intensity was evaluated as this could also affect the cells’ performance [21]. Biotransformations were also performed under low light (40–60 µmol photons m^−2^ s^−1^) to determine the effect of lower light intensity on the activity of the cells. Figure 2 shows the whole-cell biotransformation of **1a** mediated by Syn::P_*cpcB*_YqjM in light and dark conditions in the absence and in the presence of D-Glu (2.5 mM).

**Fig. 3:**
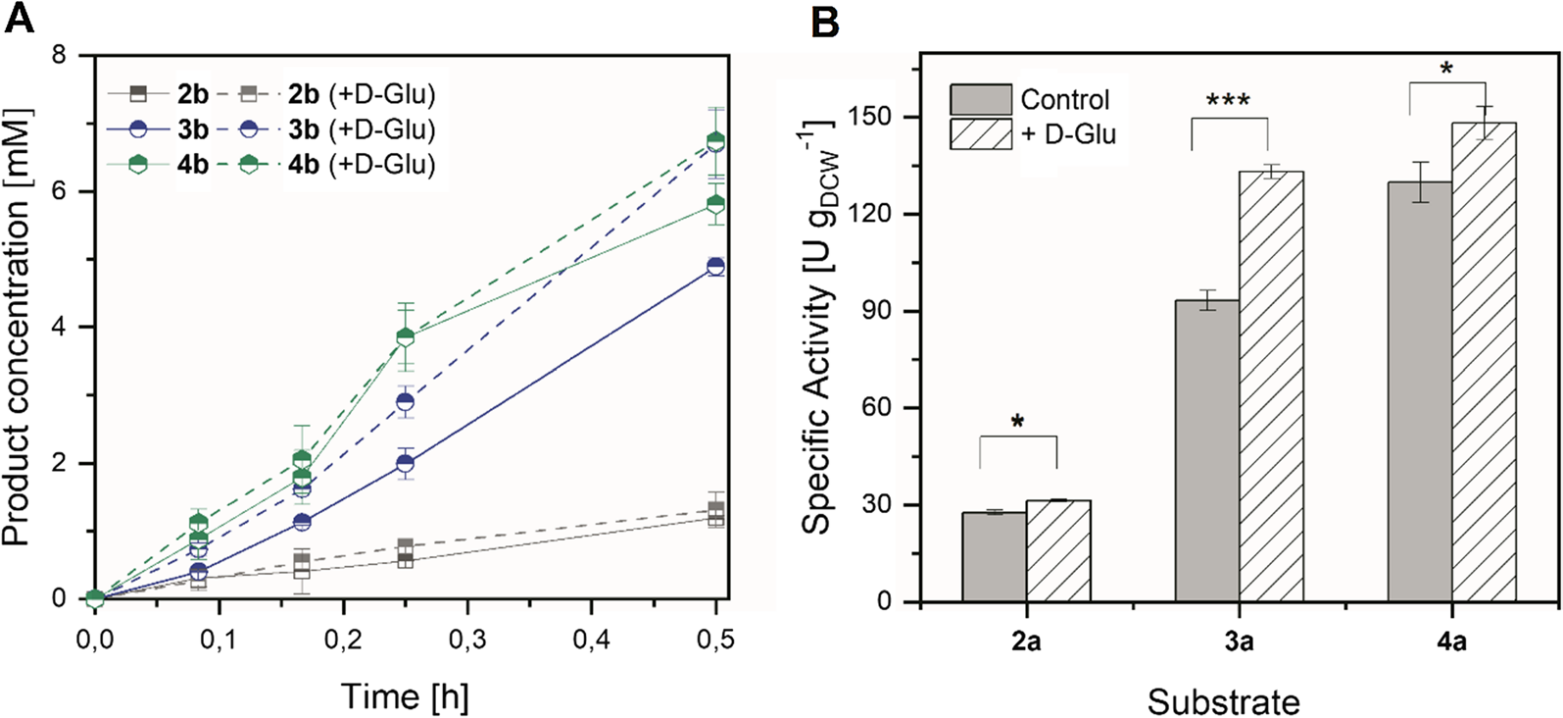
Stimulating effect of D-Glu in the reduction of various substrates mediated by Syn::P_*cpcB*_YqjM. (**A**) Time course of product formation and (**B**) Specific activities using various substrates mediated by Syn::P_*cpcB*_YqjM. *Reaction conditions*: V = 1.2 mL, T = 30 °C, 160 rpm, Initial concentration of **1a** = 10 mM, Light intensity = 200 µmol photons m^−2^ s^−1^, D-Glu = 2.5 mM (added at the start of the reaction), *N* = 3. Control reactions were performed in the absence of D-Glu. *P* values were calculated using Welch´s *t* test (*P ≤ 0.05; ***P ≤ 0.0005) and correspond to the specific activity comparison between control experiments and with D-Glu addition

As expected, the rate of the biotransformation in BG11 medium under light decreased at higher cell densities. The specific activities under dark were also improved upon D-Glu addition. The reaction proceeded in darkness with a much lower rate of approximately 20 U g_DCW_^−1^ at different optical densities (Fig. 2C). It is known that photoautotrophically grown cyanobacteria can catalyze reductions in darkness, albeit with lower rates and incomplete conversion [31]. In the absence of a reduced carbon source, the observed activity could be attributed to the degradation of storage compounds that are accumulated during the growth under photoautotrophic conditions. The constant specific activity at different cell densities is typical for biotransformations in heterotrophic organisms at a certain range until a component of the medium (ex. carbon or electron source, oxygen) becomes limiting.

We then studied the effect of D-Glu as an additional organic carbon source as well as an electron source. Indeed, the addition of D-Glu enhanced the production of **1b** for low (Fig. 2A) and high cell densities (Fig. 2B) both in light and dark conditions. We have monitored D-Glu consumption during biotransformations in the presence of **1a** under light and dark conditions (Additional file 1: Figure S1) and have observed a D-Glu decrease of 1.75 mM h^−1^ under light conditions. Specifically, a 1.5-fold increase of specific activity (150 U g_DCW_^−1^, 6.5 U mg_Chl*a*_^−1^) at an OD_750_ = 2.5 was observed upon D-Glu addition. The increase was less pronounced at higher cell densities. It should be noted that the cells used for these reactions originate from the same batch cultures and therefore should not have any differences regarding their genetic regulation.

The stimulating effect of D-Glu was also observed in the reduction of substrates **2a**–**4a** as shown in Fig. 3. In the presence of D-Glu, an increase in the specific activities for all the substrates tested was observed with **3a** exhibiting the highest increase (43%).

The specific activities shown in Fig. 2C resulted from cells grown under photoautotrophic conditions and not adapted for photomixotrophic growth. Addition of D-Glu clearly has a stimulating effect on the biotransformation. A significant increase in the specific activity in the reduction of **1a** at an OD_750_ = 2.5 was observed most especially for cells cultivated in high light (Fig. 2C). The light-driven biotransformation was considerably faster as compared to reactions in darkness with or without D-Glu. Under these conditions, the photosynthetic metabolism provides more reaction equivalents for a heterologous biotransformation than glycolytic pathways. This effect holds up to a cell density of 2.4 g_DCW_ L^−1^ (OD_750_ = 10).

Interestingly, an increase in D-Glu concentration did not lead to an increase of the initial rate (Additional file 1: Figure S2A). A twofold increase in specific activity was observed when 2.5 mM of D-Glu was supplemented. However, there was no remarkable enhancement in the activity when the sugar concentration was increased up to 12.5 mM. Additional file 1: Figure S2B showed that supplementing other sugars such as D-Galactose (D-Gal), D-Saccharose (D-Sac), D-Sorbitol (D-Sor) and D-Fructose (D-Fru) did not improve product formation rates. The efficiency of the utilization of D-Glu for the biotransformation remains unknown as it can be expected that a part of the reduced nicotinamide cofactors will be used for respiration and biomass formation and will not be dedicated to cofactor recycling.

### Increased NAD(P)H Availability in the presence of D-Glu

**Fig. 4:**
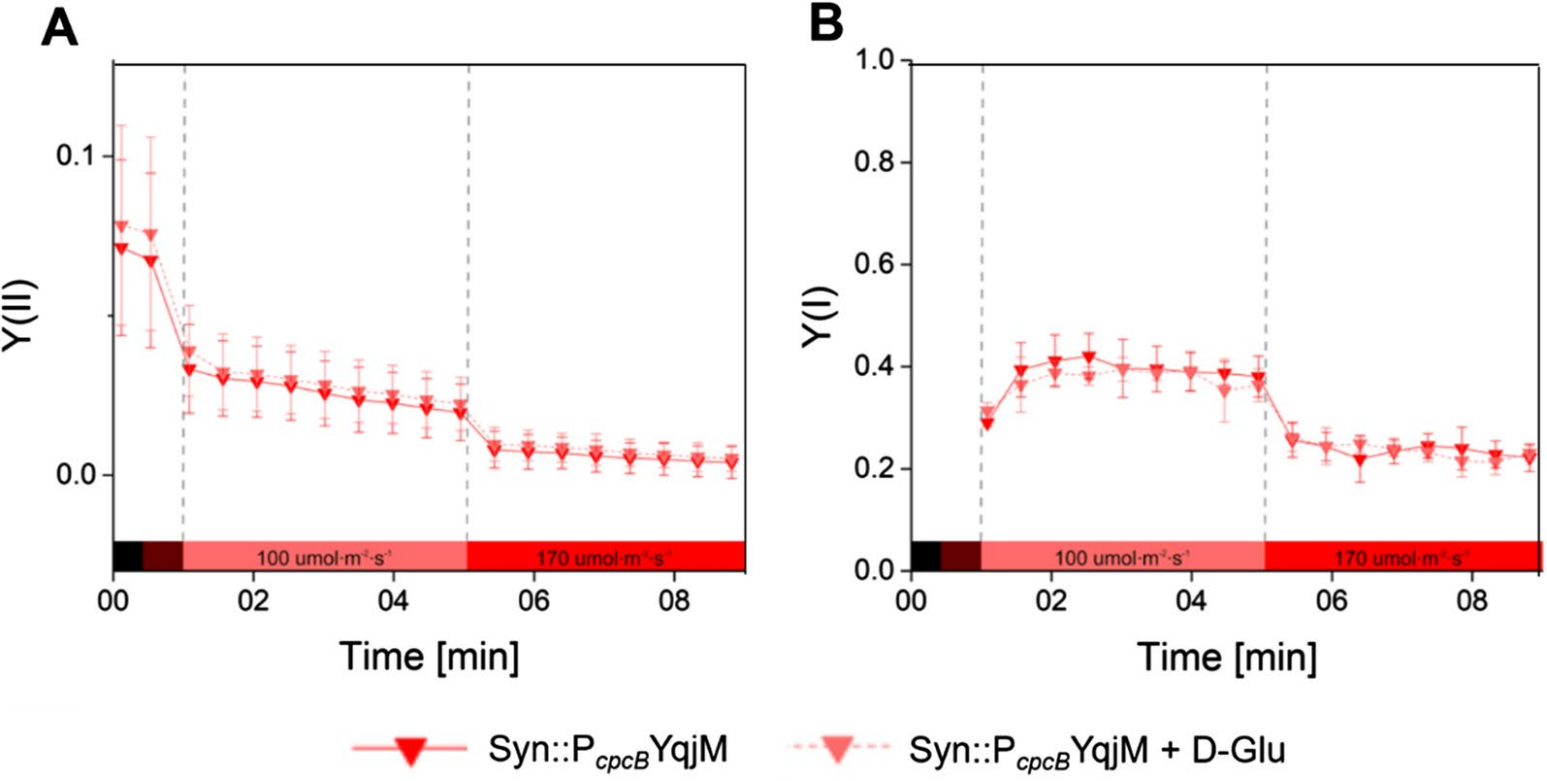
Effective yields of (**A** PSII and (**B**) PSI in the biotransformation of **1a** (10 mM) mediated by Syn::P_*cpcB*_YqjM with and without the presence of D-Glu (2.5 mM). Colored bar represents light conditions during the measurement (i.e. black = dark; dark red = far red light; light red = red light–100 µmol photons m^−2^ s^−1^; bright red = red light–170 µmol photons m^−2^ s^−1^; *N* = 3)

In order to determine if the enhanced substrate conversion rate of YqjM in the presence of D-Glu (Fig. 2) stems from increased photosynthetic electron transport, we assessed the effective yields of Photosystem II (PSII), Y(II) and Photosystem I (PSI), Y(I) in photoautotrophically grown cells with and without D-Glu (2.5 mM). While addition of D-Glu improves the productivity of YqjM (Fig. 2), our results showed no significant differences in photosynthetic yield, i.e., Y(II) or Y(I), between the samples with and without D-Glu (Fig. 4).

**Fig. 5:**
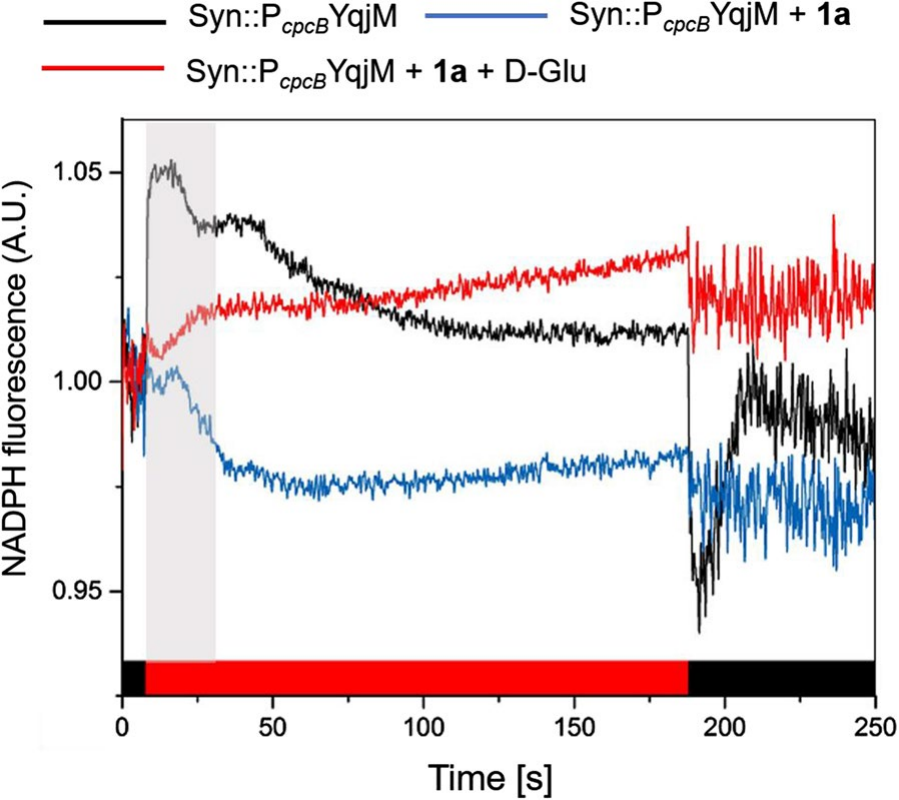
Light-induced NAD(P)H fluorescence kinetics in Syn::P_*cpcB*_YqjM. *Reaction conditions*: D-Glu = 2.5 mM, **1a** = 10 mM (added to the sample 30 min prior measurement). Cells (chl*a* = 2.5 µg mL^−1^) were placed in dark for 10 s (black bar) followed by illumination under red actinic light (200 µmol m^−2^ s^−1^, red bar) for 180 s and finally in dark for 60 s (black bar). See Materials and Methods section for the detailed procedure

Next, we monitored NAD(P)H kinetics during dark–light–dark transition (Fig. 5). Our results showed that active YqjM prevents strong light-induced reduction of the NAD(P)H/NADP^+^ pool and causes it to become even more oxidized in comparison with the dark-adapted state during the first 30 s of illumination (Fig. 5 gray panel). These findings indicate strong and rapid consumption of photosynthetically generated NAD(P)H by YqjM, supporting the idea of NAD(P)H availability as the limiting factor to YqjM activity. Addition of D-Glu causes a slow upward drift in the NAD(P)H fluorescence signal, which may be explained by NADH and NADPH supplied by increased glycolytic and OPPp activity, respectively. This could explain higher specific biotransformation rates (and yields) obtained in the presence of D-Glu. It is worth noting that NADH contributes to the NADPH fluorescence signal [32].

### Mixotrophic cultivation decreases YqjM activity

**Fig. 6:**
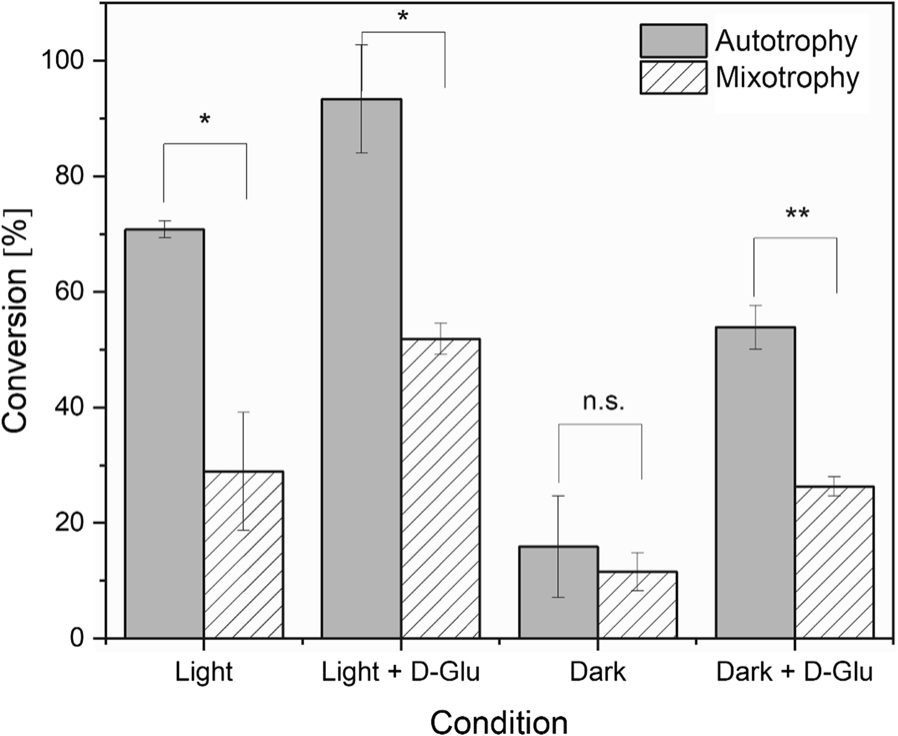
Product yields in YqjM-mediated biotransformation of **1a** using Syn::P_*cpcB*_YqjM grown 48 h under photoautotrophy and photomixotrophy (D-Glu, 5 mM). The yield was obtained after 30 min of the reaction. Cells were grown under high light (200 µmol photons m^−2^ s^−1^). *Reaction conditions*: V = 1.2 mL, T = 30 °C, 160 rpm, Initial concentration of **1a** = 10 mM, Light intensity = 200 µmol photons m^−2^ s^−1^, DCW = 2.4 g L^−1^, *N* = 3. *P* values were calculated using *t* test (*P ≤ 0.05; **P ≤ 0.005; *n.s.* = not significant)

While the use of the promoter P_*cpcB*_ allows obtaining very high rates, this promoter might be differentially regulated under different cultivation conditions. To investigate this, we cultivated cells in the presence of D-Glu (5 mM) for 48 h prior to the biotransformation. Intracellular in vitro YqjM activity was lower when cells were grown under photomixotrophic conditions as compared to cells grown photoautotrophically (Additional file 1: Figure S3). Mixotrophically grown cells also showed lower conversions after 30 min in the whole-cell biotransformations of **1a** (Fig. 6). Nevertheless, for both cultivation conditions, addition of D-Glu accelerated the reaction. Interestingly, biotransformation in darkness with D-Glu resulted to a similar level of conversion as that of reactions performed under light without D-Glu. Apart from reactions performed in darkness without D-Glu addition, significant improvements in conversion were observed for other reaction systems (Fig. 6).

### Addition of glucose also enhances YqjM activity in scaled-up systems

**Fig. 7:**
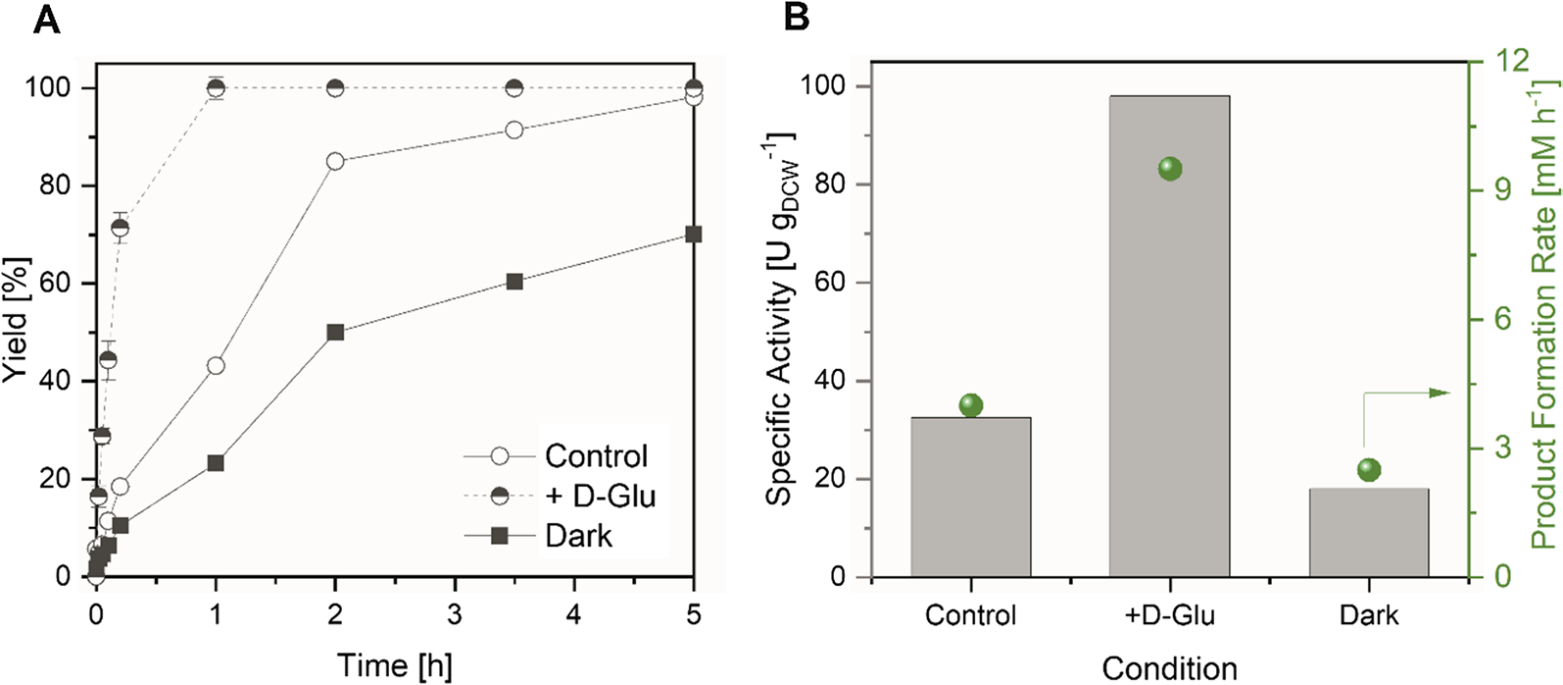
Whole-cell biotransformation of **1a** mediated by Syn::P_*cpcB*_YqjM performed in an internally illuminated BCR. (**A**) Yield of **1b** during the course of the reaction and (**B**) Specific activities relative to cell dry weight and product formation rate at various conditions. *Reaction conditions:* V= 200 mL, T= 30 °C, air flow rate= 0.6 L min^-1^, initial concentration of **1a** = 10 mM, number of WLE’s = 40; DCW = 2.4 g L^-1^, *N*=1–2

After characterization of the effect of D-Glu in small scale, we studied the catalytic rates in a 200 mL reaction volume in an internally illuminated Bubble Column Reactor (BCR) (Fig. 7). We were pleased to find that addition of D-Glu in this system remarkably increased the volumetric productivity in the formation of **1b** by 2.4-fold. The substrate **1a** was fully converted within one hour, approximately two times faster than with internal illumination in the absence of D-Glu [19].

## Discussion

Here, we have demonstrated that the addition of sugar particularly D-Glu could improve the productivity of *Synechocystis* cells expressing the ene-reductase YqjM from *B. subtilis*. This was also corroborated by NAD(P)H fluorescence measurements showing an increased fluorescence (i.e. increased supply) upon D-Glu addition. Moreover, we have also shown that the system is also applicable in larger volumes using the internally illuminated BCR. Oxidoreductases expressed in cyanobacteria represent a strong electron drain which allows us to investigate how much of the electrons (i.e. originating from the photosynthetic electron transport chain) can be deviated towards heterologous biotechnological processes, ranging from the production of high-value products to bulk chemicals and even biofuels. The stereoselective reduction of **1a** by the ene-reductase YqjM has been the fastest recorded photobiotransformation so far with specific activities of over 100 U g_DCW_^−1^ in *Synechocystis* [12, 21]. Stopped-flow kinetics indicated that the maximal turnover rate of the oxidative half reaction of YqjM for the reduction of **1a** is more than 50 times higher than the reductive half reaction (i.e. the oxidation of NADPH). This means that in the presence of **1a** the enzyme will be constantly in the oxidized state. Furthermore, YqjM has a *K*_D_ value of 39.8 µM towards NADPH. On the other hand, a *K*_D_ value of 140.4 µM was determined for NADH. A concentration of 500 µM is required to reach saturation and any drop below 200 µM will strongly reduce the turnover rate making this enzymatic reaction NAD(P)H-limited [21]. Recently, Tanaka et al. reported a concentration of 87.4 nM OD_730_^−1^ for the intracellular NADPH concentration in photoautotrophically grown *Synechocystis* which is below this benchmark value [33].

Nakamura et al. reported that the reduction of 2A,3A,4A,5A,6A-pentafluoroacetophenone and other prochiral ketones in *Synechococcus elongatus* PCC 7942 by endogenous ketoreductases proceeds not only under light but also in darkness [31] and in the presence of 3-(3,4-dichlorophenyl)-1,1-dimethylurea (DCMU), a specific inhibitor of the water-splitting PSII, albeit both with significantly lower rates than under light. This finding shows that glycolytic pathways in cyanobacteria can sustain heterologous NAD(P)H-consuming redox reactions, albeit at lower rates than under light. In this work, the stimulating effect of D-Glu addition was observed at different cell densities and was independent from the light intensity during the cultivation and the choice of the substrate. This demonstrates that the biotransformation can be supplied with electrons originating from glycolytic pathways. Addition of D-Glu to the reaction under light led to a 50% increase of the rate at a cell dry weight of 0.6 g L^−1^. This effect was observed under all the tested cell densities, most significantly at lower cell densities. At low cell densities with minimal self-shading of the cells, an improvement of the electron supplies by D-Glu addition boosts product formation.

The enzymes involved in the OPPp are expressed under photoautotrophic growth, interacting with the Calvin cycle [30, 34]. It should be noted that we applied cells for biotransformations with the metabolism adapted for autotrophic growth under high light (e.g. 180–200 µmol photons m^−2^ s^−1^), where some genes from the glycolytic pathways are less expressed [28, 35, 36]. The flux level of the OPPp is higher under the light intensity of 40 µmol m^−2^ s^−1^ than 125 µmol m^−2^ s^−1^ under mixotrophic conditions. The gluconeogenic activity upstream of glycolysis, probably caused by the *gap1* gene repression enhanced the flux of the OPPp [28]. In the earlier light period, the following genes coding for OPPp enzymes have been observed as upregulated: *tktA* (*sll1070*), *devB* (*sll1479*), and *cfxE* (*sll0807*). In the later light period, the upregulation of *rpiA* was noted [*slr0194*, encoding the ribose 5-phosphate isomerase (RPI)] [34]. Nevertheless, the stimulating effect of D-Glu was also observed after cultivation at lower light intensity. Under mixotrophic conditions, the enzymes of the pentose phosphate cycle are down-regulated, which might contribute to the lower whole-cell activity observed after cultivation under light and addition of D-Glu to the medium [30].

Interestingly, the enhanced YqjM production rate in the presence of D-Glu is not caused by an increase in the photosynthetic electron transport rate. We hypothesize that under the photoautotrophic conditions, the reaction is electron-limited, which is mitigated by the metabolized D-Glu. These findings are in line with the observation that whole-cell biotransformations led to a decrease of intracellular NAD(P)H levels [21]. Addition of D-Glu may increase the amount of NADH and NADPH available to YqjM, but the extra electrons are likely derived from glycolysis and OPPp. Still, a light-dependent component in the D-Glu-induced enhancement of YqjM-catalyzed substrate conversion was observed (Fig. 2), suggesting involvement of a light-induced regulatory mechanism. It should be mentioned that **1a** is a thiol-reactive compound that potentially disturbs the thiol-based regulatory mechanisms in the cell, such as the thioredoxin and glutaredoxin systems. Indeed, glycolysis, the TCA cycle, the Calvin–Benson–Bassham cycle (CBB), and OPPp, all of which profoundly affect the NADPH/NADH metabolism, are likely subject to light-dependent redox regulation [37]. Nevertheless, a significant activating effect of D-Glu was also observed with **4a** a substrate that does not have this side-reactivity.

The possibility to boost redox reactions under light with sugar addition also implies that the initial rates observed under photoautotrophic conditions might be partially fuelled by the catabolism of storage compounds. As glycolytic pathways are usually regulated on a metabolic level by the ratio of reduced and oxidized nicotinamide cofactors, we assume that lower NAD(P)H levels increase the activity in these pathways. Several factors are involved upon the addition of D-Glu. The breakdown of storage compounds may be to some extent light dependent. Taking into account that in plant chloroplasts the degradation of starch can be activated by light especially during stressful conditions [38] as such as the high light utilized during our study.

The rate increase caused by D-Glu addition was also observed in a 200 mL internally illuminated photobioreactor, leading to a volumetric productivity as high as 10 mM h^−1^. This demonstrates that biotransformations fuelled by a combination of photosynthesis and carbohydrate catabolism can also be exploited for biotechnological production.

Our results showed that under photoautotrophic conditions, the ene-reductase is not NAD(P)H-saturated and has a higher capacity for substrate conversion. Cyanobacteria harboring heterologous enzymes such as monooxygenases and oxidoreductases have already been employed for the production of targeted chemicals and value-added compounds. Specific activities in the range of 5–6 U g_DCW_^−1^ have been reported for monooxygenases [13, 39] while higher activities have been reported for oxidoreductases ranging from 20 to over 100 U g_DCW_^−1^ [15, 21]. Moreover, biotransformations using Baeyer–Villiger monooxgenases report specific activities ranging from 25 to 60 U g_DCW_^−1^ [16, 17]. These works provide proof-of-concept, but also show that the rates obtained in photoautotrophic microorganisms are not higher than rates obtained in heterotrophs. This raises the question on how cyanobacteria might be engineered to sustain processes for the production of chemicals and biofuels with much higher electron consumption. In this context, some recent works indicate the possibility to engineer the cyanobacterial metabolism for an increased electron supply: deletion of flavodiiron proteins (FDPs) as competing electron sinks increased the specific activities of ene-reductions [21] and Baeyer–Villiger oxidations [17] in *Synechocystis*. By expressing heterologous metabolic sinks in cyanobacteria, an improved photosynthetic efficiency and performance was reported [40]. Moreover, by inactivation of NDH-1, electron flow was increased by 30% to a heterologous cytochrome P450 expressed in *Synechococcus* PCC 7002 [41].

In conclusion, the addition of sugar specifically D-Glu during a photobiotransformation by an ene-reductase in *Synechocystis* improved the specific activity without an increase of the photosynthetic activity, indicating that electron-consuming processes at a specific activity of 100 g_DCW_ L^−1^ are already electron limited. The higher activity can be attributed to an increase of NAD(P)H. As glycolytic pathways lead to the reduction of NADPH and NADH, and the enzyme accepts electrons from both nicotinamide cofactors, it is not possible to assign the activity increase to either one of them. A further increase of the yields of cyanobacterial processes thus requires a substantial improvement by cell engineering. Nevertheless, several recent works demonstrated the feasibility of such an optimization, which opens an avenue toward much higher productivity in the photosynthetic production of biofuels and bioproducts.

## Materials and methods

### General

The substrate, **1a** has been synthesized as previously described [12]. The product, **1b** was received as a white powder from Chiracon (Luckenwalde, Germany), Sigma-Aldrich (Steinheim, Germany). Other chemicals were procured from Roth (Krems, Austria) or Sigma-Aldrich (Steinheim, Germany). The dry cell weight (DCW) was previously determined through lyophilization of the cell suspensions at different OD_750_ values (i.e. OD_750_ of 10 corresponds to 2.4 g_DCW_ L^−1^) [21].

### Strains and growth conditions

*Synechocystis* strains (e.g., wild type and mutant) utilized in this study are listed in Additional file 1: Table S1. Strains were cultured under two light conditions in standard BG11 medium. For low light cultivation, liquid seed cultures were placed in a plant growth chamber (SWGC-1000, WISD lab instruments) under continuous white light illumination (light intensity = 40–60 μmol photons m^−2^ s^−1^) and placed on rotary shakers (140 rpm) incorporated inside the chamber. The temperature and humidity of the chamber were maintained at 30 °C and 50%, respectively. For high light cultivations, liquid inoculums were placed under a tunable LED lamp (CellDEG, Berlin, Germany) emitting red and blue light (light intensity = 200 μmol photons m^−2^ s^−1^). Cultures were agitated at 160 rpm and placed inside a chamber (Thermo Electro Corporation) maintained at 30 °C. Kanamycin (50 μg mL^−1^) was added as a selective marker to mutant strains containing the P_*cpcB*_::*yqjM* cassette. Liquid inoculums with a working volume of either 100 mL or 150 mL were prepared and allowed to grow until an OD_750_ = 1–2. The cells were cultivated photoautotrophically or photomixotrophically (i.e. light and addition of 5 mM D-Glu unless otherwise indicated) during inoculation. D-Glu at a concentration of 2.5 mM was added at the start of the biocatalytic reaction.

### Whole-cell biotransformations and analytics

Cells were harvested and concentrated by centrifugation (RT, 15 min, 3220* g*) after reaching an OD_750_ = 1–2 which typically takes 4–5 days of cultivation. The pellet was re-suspended in fresh BG11 and adjusted to the desired optical density. The reaction was initiated by addition of the substrate at a concentration of 10 mM. Samples were taken at several time points and quenched in liquid nitrogen. The in vitro NADPH Assay was performed as previously described [21]. The protein content of the cell-free extracts (CFE) was determined with the BCA Assay. The D-Glu content was determined using the Sucrose and Glucose Assay Kit from Sigma-Aldrich.

### Conditions applied during in vivo biocatalysis with cyanobacteria

Organic carbon sources such as D-Glu (2.5, 5, 7.5, 10, and 12.5 mM), D-Gal (10 mM), D-Sac (10 mM), D-Sor (10 mM) and D-Fru (10 mM) have been added together with the substrate at the beginning of the reaction. Reactions with D-Glu (5 mM) which was added during cultivation were also performed.

### Quantification of substrates and products using Gas Chromatography-Flame Ionization Detector (GC–FID)

Aliquots (100 µL) from the reaction mixture using substrates **1a**–**3a** and their corresponding products were extracted by the addition of 300 µL ethyl acetate with 2 mM *n*-decanol as internal standard. The organic layer was dried using one spatula tip of anhydrous MgSO_4_, centrifuged and analyzed using Gas Chromatography Flame Ionization Detector (GC–FID, GC-2010 Plus, Shimadzu, Japan) fitted with a ZB-5 column (Macherey–Nagel, Düren, Germany). For **4a** and **4b**, dichloromethane containing 2 mM of *n*-decanol was used instead of ethyl acetate. Nitrogen was used as the carrier gas with a split ratio of 20. The parameters for the GC–FID methods for all the substrates and products were chosen as described previously [21].

### Photo-biotransformations in the Bubble Column Reactor (BCR)

Upon reaching an OD_750_ = 1–2, cells were harvested by centrifugation (24 °C, 20 min, 3220* g*) and inoculated in gas washing tubes (working volume of 200 mL) at an OD_750_ = 0.1 supplemented with Kanamycin (50 µg mL^−1^). The tubes were then placed in an aquarium maintained at 30 °C and illuminated using six fluorescent lamps delivering a light intensity of 200–250 μmol photons m^−2^ s^−1^. After reaching an OD_750_ = 1–2, cells were further harvested and centrifuged. The pellet was re-suspended in an appropriate volume of BG11 to OD_750_ = 10 and subsequently utilized for whole-cell biotransformations in the BCR (*φ*_i_ = 5 cm, h = 50 cm, V = 200 mL) fitted with emitting coils. Wireless Light Emitters (WLEs, 40 pieces) emitting white light were suspended in the reactor to provide internal illumination. The WLEs consist of a white LED plus a receiving coil inside a polycarbonate shell as described previously [19]. The air was supplied via an electric pump (Boyu S-4000B) at a rate of 0.6 L min^−1^. A fan was utilized to maintain the temperature at 30 °C positioned 5 cm from the reactor. D-Glu (5 mM) was added in parallel with **1a** (10 mM) to initiate the reaction. Aliquots (100 μL) were taken at different time points, quenched in liquid nitrogen and stored at −20 °C prior analysis with GC–FID.

### Quantification of the chlorophyll *a* content

The content of chlorophyll *a* (chl*a*) was determined as previously reported [21]. Briefly, the pellet of a 100 µL sample was re-suspended in 100 µL of ddH_2_O followed by addition of 900 µL methanol. The samples were shortly vortexed and incubated in darkness for a maximum of 10 min. The absorbance was then read at 665 nm and an extinction coefficient of 78.74 L g^−1^ cm^−1^ was used to determine the amount of chl*a*.

### Determination of Photosynthetic Yields

The pulse amplitude-modulated spectrophotometer DUAL-PAM-100 (Walz, Germany) was used to measure chlorophyll fluorescence and P700 redox kinetics. Prior measurement, the cells were adjusted to OD_750_ = 2.5 and exposed to illumination of 200 μmol m^−2^ s^−1^ for 30 min in the presence of **1a** (10 mM). When applicable, D-Glu (2.5 mM) was added simultaneously with the substrate. Before the measurement, samples were dark-adapted for 2 min. During the measurement, multiple turnover saturating pulses (5000 μmol m^−2^ s^−1^, 500 ms) were applied under continuous actinic red illumination (100 and 170 μmol m^−2^ s^−1^). The effective yields of PSI and PSII [Y(I) and Y(II), respectively] were calculated by the DUAL-PAM software.

### Light-induced NAD(P)H Redox kinetics

NAD(P)H fluorescence was measured with the NADPH/9-AA module for DUAL-PAM-100 (Walz, Germany). Cells were harvested and adjusted to OD_750_ = 2.5 and exposed to illumination of 200 μmol m^−2^ s^−1^ for 30 min in the presence of **1a** (10 mM). When applicable, D-Glu (2.5 mM) was added simultaneously with the substrate. Afterward, samples were dark-adapted for 15 min and diluted to reach a chl*a* concentration of 2.5 µg mL^−1^. The following protocol was utilized: dark (10 s)–actinic red (180 s, 200 μmol m^−2^ s^−1^)–dark (60 s).

### Statistical analysis

Analyses were performed using GraphPad Prism version 8.0. The analysis of two groups were assessed using unpaired Welch’s *t* test. Assumption of normality was assessed using Shapiro–Wilk test. Data stemmed from at least 3 biological replicates.

## Supplementary Information

**Additional file 1: Table S1.** Strains and Plasmids used in this study. **Figure S1.** Biotransformation of **1a** catalysed by Syn::P_*cpcB*_YqjM in the presence of D-Glu (2.5 mM) in (**A**) Light and (**B**) Dark. D-Glu was added together with **1a** to initiate the reaction and monitored throughout the course of the biotransformation. Reaction conditions: V= 1.2 mL, T= 30 °C, 160 rpm, Initial concentration of **1a** = 10 mM, Light intensity = 200 μmol photons m^-2^ s^-1^, DCW = 2.4 g L^-1^, *N*= 2. **Figure S2.** (**A**) Specific activities in the biotransformation of **1a** mediated by Syn::P_*cpcB*_YqjM in the presence of various concentration of D-Glu and (**B**) Time course production of **1b** from the reduction of **1a** in the presence of different sugars. *Reaction conditions*: *V*= 1.2 mL, T= 30 °C, 140 rpm, Initial concentration of **1a** = 10 mM, Light intensity = 40–60 μmol photons m^-2^ s^-1^, DCW = 2.4 g L^-1^, *N*= 3. Sugars were added at a concentration of 10 mM together with **1a** to initiate the reaction. Control reactions were performed without addition of any sugars. **Figure S3.** Specific activities (*in vitro*) of YqjM in the biotransformation of **1a**. Cells were cultivated in the presence of D-Glu (5 mM) for 48 h under mixotrophic conditions. Standard cultivation conditions in BG-11 were performed for autotrophic conditions. *N*=3.

## Data Availability

All data generated or analyzed during this study are included in this published article and its Additional files.

## Abbreviations

**1a**: 2-Methylmaleimide
**1b**: 2-Methylsuccinimide
**2a**: *N*-Methylmaleimide
**2b**: *N*-Methylsuccinimide
**3a**: 2-Methyl-*N*-Methylmaleimide
**3b**: 2-Methyl-*N*-Methylsuccinimide
**4a**: 2-Cyclohexen-1-one
**4b**: Cyclohexanone
BCR: Bubble Column Reactor
DCMU: 3-(3,4-Dichlorophenyl)-1,1-dimethylurea
DCW: Dry Cell Weight
d-Glu: d-Glucose
ED: Entner–Doudoroff pathway
EMP: Embden–Meyerhof–Parnas pathway
OPPp: Oxidative pentose phosphate pathway
PSI: Photosystem I
PSII: Photosystem II
*Synechocystis*/Syn: *Synechocystis* sp. PCC 6803
NADH: Nicotinamide adenine dinucleotide
NADPH: Nicotinamide adenine dinucleotide phosphate
WLE: Wireless Light Emitters
Y(I): Effective yield of Photosystem I
Y(II): Effective yield of Photosystem II
