# Supplementary material for: Towards the rate limit of heterologous biotechnological reactions in recombinant cyanobacteria
Source: Biotechnol Biofuels Bioprod. 2023 Jan 6;16:4. doi: 10.1186/s13068-022-02237-4 (PMC9825001; doi:10.1186/s13068-022-02237-4)
Supplement: Supplementary file 1 — Additional file 1: Table S1. Strains and Plasmids used in this study. Figure S1. Biotransformation of 1a catalysed by Syn::PcpcBYqjM in the presence of D-Glu (2.5 mM) in (A) Light and (B) Dark. D-Glu was added together with 1a to initiate the reaction and monitored throughout the course of the biotransformation. Reaction conditions: V= 1.2 mL, T= 30 °C, 160 rpm, Initial concentration of 1a = 10 mM, Light intensity = 200 μmol photons m-2 s-1, DCW = 2.4 g L-1, N= 2. Figure S2. (A) Specific activities in the biotransformation of 1a mediated by Syn::PcpcBYqjM in the presence of various concentration of D-Glu and (B) Time course production of 1b from the reduction of 1a in the presence of different sugars. Reaction conditions: V= 1.2 mL, T= 30 °C, 140 rpm, Initial concentration of 1a = 10 mM, Light intensity = 40–60 μmol photons m-2 s-1, DCW = 2.4 g L-1, N= 3. Sugars were added at a concentration of 10 mM together with 1a to initiate the reaction. Control reactions were performed without addition of any sugars. Figure S3. Specific activities (in vitro) of YqjM in the biotransformation of 1a. Cells were cultivated in the presence of D-Glu (5 mM) for 48 h under mixotrophic conditions. Standard cultivation conditions in BG-11 were performed for autotrophic conditions. N=3. [file 13068_2022_2237_MOESM1_ESM.docx]

**Supporting Information**

**Towards the rate limit of heterologous biotechnological reactions in recombinant cyanobacteria**

**Giovanni Davide Barone^1,2,3^, Michal Hubáček^4^, Lenny Malihan-Yap^1^, Hanna C. Grimm^1^, Lauri Nikkanen^4^, Catarina Pacheco^2^, Paula Tamagnini^2,3^, Yagut Allahverdiyeva^4^, and Robert Kourist^1*^**

^1^Biocatalysis and Protein Engineering, Institute of Molecular Biotechnology, Graz University of Technology, Graz, 8010, Austria

^2^i3S - Instituto de Investigação e Inovação em Saúde, IBMC - Instituto de Biologia Molecular e Celular, Universidade do Porto, Porto, 4200-135 Porto, Portugal

^3^Departamento de Biologia Faculdade de Ciências, Universidade do Porto Rua do Campo Alegre, Edifício FC4, 4169-007 Porto, Portugal

^4^Laboratory of Molecular Plant Biology, Department of Life Technologies, University of Turku, Turku FI–20014,

Finland

^*^[kourist@tugraz.at](mailto:kourist@tugraz.at)

**Table S1.** Strains and Plasmids used in this study.

| **Strains** | **Description** | **Reference** |
| --- | --- | --- |
| *Synechocystis* sp. PCC 6803 Wild Type | Wild Type *Synechocystis* sp. PCC 6803 | [1] |
| SynRekB_P*_cpcB_*YqjM | Integrative plasmid with a P*_cpcB_* promoter harboring the ene-reductase from *Bacillus subtilis*, His-tag (N) | [2] |
| *Synechocystis* sp. PCC 6803 P*_cpcB_*::YqjM | *Synechocystis* sp. PCC 6803 harboring the YqjM gene from *Bacillus subtilis* | [2] |


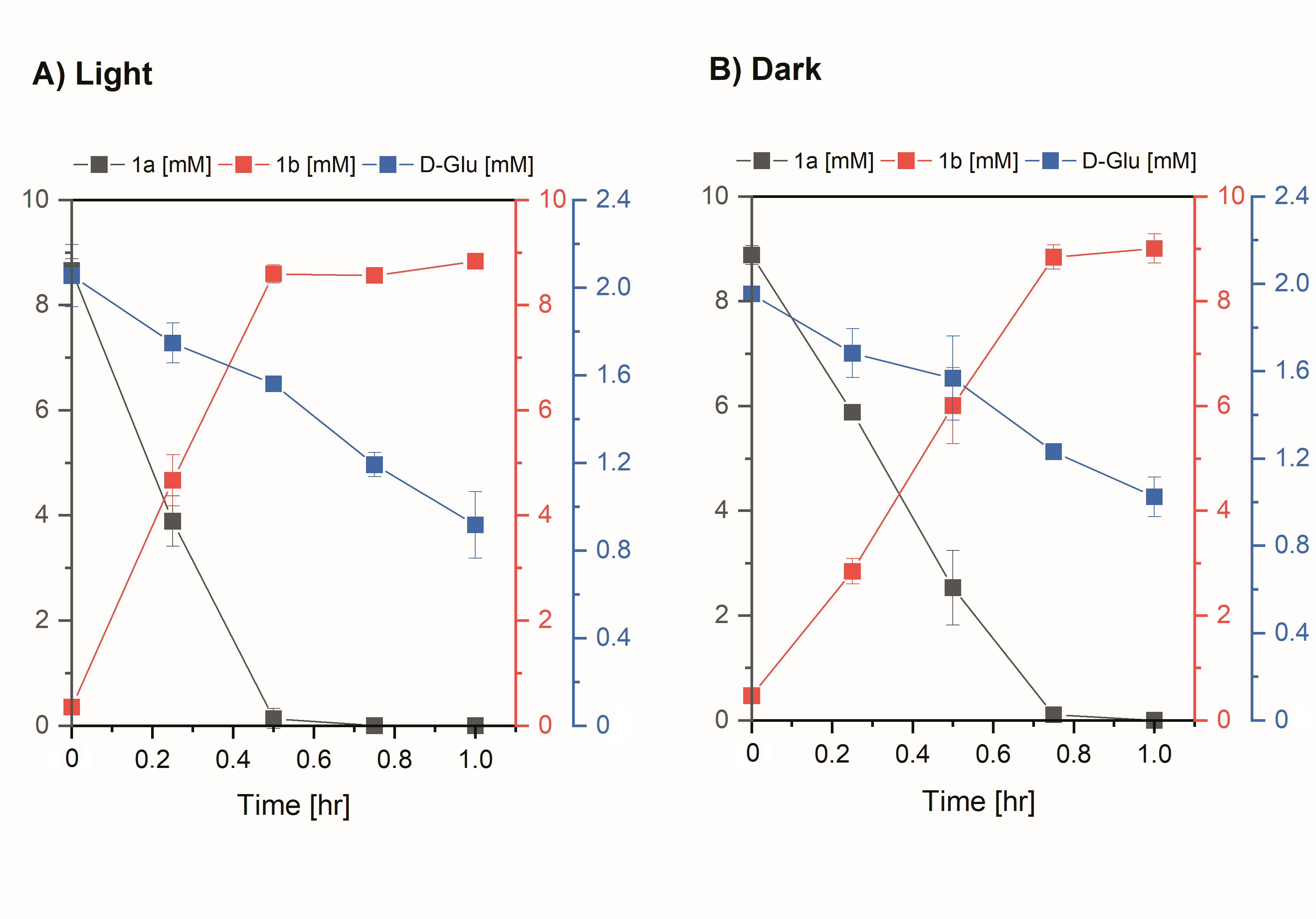


**Figure S1.** Biotransformation of **1a** catalysed by Syn::P*_cpcB_*YqjM in the presence of D-Glu (2.5 mM) in (A) Light and (B) Dark. D-Glu was added together with **1a** to initiate the reaction and monitored throughout the course of the biotransformation. *Reaction conditions:* V= 1.2 mL, T= 30 °C, 160 rpm, Initial concentration of **1a** = 10 mM, Light intensity = 200 µmol photons m^-2^ s^-1^, DCW = 2.4 g L*^-1^*, *N*= 2.


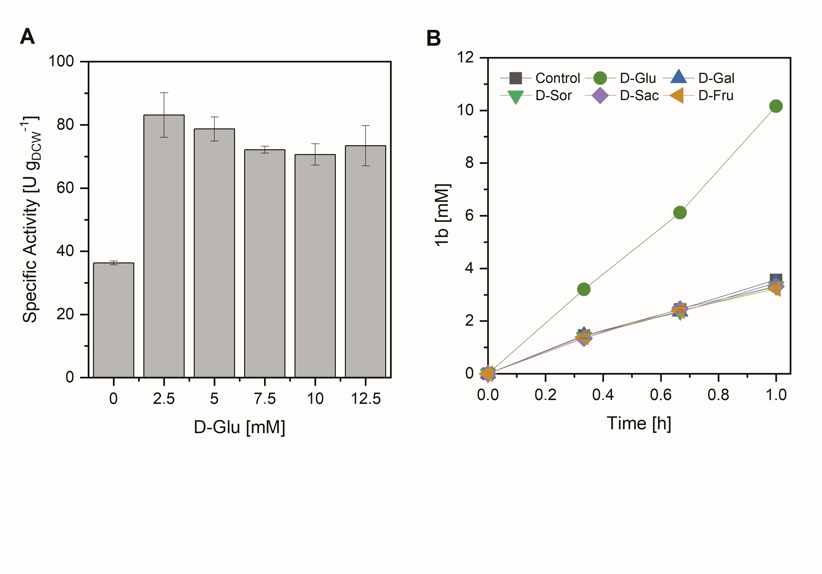


**Figure S2.** (A) Specific activities in the biotransformation of **1a** mediated by Syn::P*_cpcB_*YqjM in the presence of various concentration of D-Glu and (B) Time course production of **1b** from the reduction of **1a** in the presence of different sugars. *Reaction conditions*: V= 1.2 mL, T= 30 °C, 140 rpm, Initial concentration of **1a**= 10 mM, Light intensity = 40-60 µmol photons m^-2^ s^-1^, DCW = 2.4 g L*^-1^*, *N*= 3. Sugars were added at a concentration of 10 mM together with **1a** to initiate the reaction. Control reactions were performed without addition of any sugars.


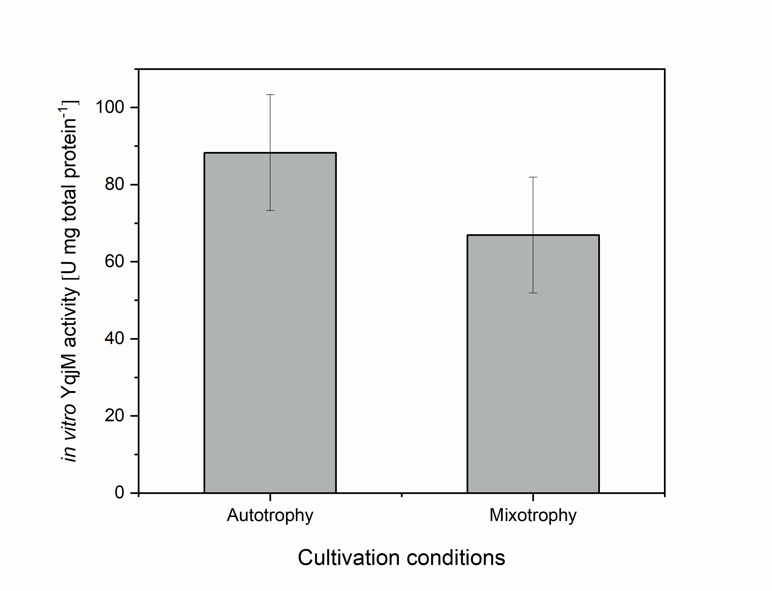


**Figure S3**. Specific activities (*in vitro*) of YqjM in the biotransformation of **1a**. Cells were cultivated in the presence of D-Glu (5 mM) for 48 h under mixotrophic conditions. Standard cultivation conditions in BG-11 were performed for autotrophic conditions. *N*= 3.

**References**

1. Stanier RY, Kunisawa R, Mandel M, Cohen-Bazire G. Purification and properties of unicellular blue-green algae (order *Chroococcales*). *Bacteriol Rev.* **35**, 171-205 (1971).

2. Assil-Companioni L, Büchsenschütz HC, Solymosi D, Dyczmons-Nowaczyk NG, Bauer KKF, Wallner S, et al. Engineering of NADPH Supply Boosts Photosynthesis-Driven Biotransformations. *ACS Catal.* **10**, 11864-77 (2020).
